# A New Efficient Digital Image Encryption Based on Inverse Left Almost Semi Group and Lorenz Chaotic System

**DOI:** 10.3390/e20120913

**Published:** 2018-11-30

**Authors:** Irfan Younas, Majid Khan

**Affiliations:** 1 Department of Mathematics, Quaid-i-Azam University, Islamabad 44000, Pakistan; 2 Department of Applied Mathematics and Statistics, Institute of Space Technology Islamabad, Islamabad 44000, Pakistan; mk.cfd1@gmail.com or; 3 Cyber and Information Security Lab, Institute of Space Technology Islamabad, Islamabad 44000, Pakistan

**Keywords:** inverse left almost semigroup, confusion and diffusion, chaotic systems, statistical analysis

## Abstract

In this research article, we propose a new structure namely inverse left almost semigroup (LA-semigroup) by adding confusion in our proposed image encryption scheme along with discrete and continuous chaotic systems in order to complement the diffusion characteristics. The performance analysis was conducted in terms of the correlation analysis, the pixels uniformity analysis, the sensitivity analysis, the information entropy analysis, and pixels difference based measurements. The results show that the proposed algorithm has better information security properties, which can provide strong security and a high performance.

## 1. Introduction

The existing world demands fast and secure communication. The bandwidth is increasing every day due to third generation, fourth generation and fifth generation technologies. Digital contents can be easily accessed through any geographical remote area because of the ideology of a global village. By advancing the technologies, smart and simple to access information from any remote station creates a massive insecurity of digital information. The security of digital multimedia is one of the vital problems of the information sciences. The widespread broadcasting and distribution of digital information over the internet, especially social media like Facebook and Twitter, have made it important to protect our most important information from theft, illegal copying, handling and sharing. The advancement of digital media technology and other multimedia related technology has made it possible to perform some standard procedures in order to improve the security of our digital images/audio/videos from being utilized criminally over the web.

There are a number of information security techniques that have been designed whose algorithms are based on cryptography, which is one of the most important branch of cryptology that is used to make an encryption scheme to secure information. Classical encryption techniques usually utilize either substitution or permutation to develop a cryptosystem; for instance, mono-alphabetical and polyalphabetic ciphers. The use of only substitution or permutation while developing any encryption scheme is susceptible to different cryptographic attacks. Consequently, the cryptosystems are getting weaker every day due to the advancement of technologies and computational powers of machines. In 1949, Claude Shannon gave the idea of confusion and diffusion, which completely changed the security mechanisms of the digital world. The information theory added a new pillar in information security. After the theory of Claude Shannon, the idea of confusion and diffusion was extensively used, which means substitution and permutation were utilized at a time in modern block ciphers [1,2]. According to Shannon, confusion specifies the connections between the cipher text and the key as much as complex whereas diffusion corresponds to the redundancy in the statistical data of the plaintext used in the statistical data of the cipher text. This master idea fundamentally changed the ideology of modern block and steam ciphers. The ideas of confusion and diffusion were utilized in a number of modern block ciphers namely international data encryption standard (IDEA), data encryption standard (DES), and advanced encryption standard (AES).

Thereafter, different new techniques were designed which use chaos theory, wavelets transform, discrete transforms, optics, DNA sequences and quantum spinning and rotations [3,4,5,6,7,8,9,10,11,12,13,14,15,16,17,18,19,20,21,22,23,24,25,26,27,28,29,30,31,32,33,34]. In recent times, chaos theory has been used in an extensive way for the development of image encryption mechanisms [35,36]. The three fundamental characteristics of chaos that have made it possible to use it in the development of encryption algorithms are sensitive to the initial condition, topological mixing, and dense periodic orbits. These three properties were closely related to cryptography. Due to the cryptographically robust characteristics of chaos, we have utilized the Lorenz chaotic system while designing our novel image encryption technique.

So far, different types of mathematical structures were utilized namely Group, Ring, Galois field and Galois ring for the construction of a substitution box (S-box), which is one of the most important nonlinear components of any modern block ciphers. The thrust of new mathematical structures for the development of encryption techniques is one of the most important areas of research in information security [8,11,12,15]. An algebraic structure equipped with a closed and left invertive binary operation is called a left almost semigroup (abbreviated as LA-semigroup). This notion was made known by Kazim and Naseeruddin in the early 1970s [37]. Mushtaq and Yusuf discussed some important properties in [38]. Such groupoids are also called right modular groupoids or left invertive groupoids or incorrectly as Abel Grassmann’s groupoid [39,40,41]. By successive applications of the left invertive law in an arbitrary LA-semigroup, it can be seen that the medial identity (st)(uv)=(su)(tv) naturally holds in an LA-semigroup. It is important to mention here that every LA-semigroup is always medial but its converse is not true. It is a non-associative and non-commutative structure midway between a groupoid and commutative semigroup.

In order to define the associative powers of elements in an LA-semigroup the identity *(ss)s = s(ss)* was introduced in Reference [42]. An LA-semigroup with this additional property is called a locally associative LA-semigroup. Some important decompositions of locally associative LA-semigroups were also investigated in References [42,43]. Consider a locally associative LA-semigroup defined by the Table 1.

Where 0∗(0∗(0∗0))=2≠1=(0∗(0∗0))∗0, substantiates that a locally associative LA-semigroup not need to have associative powers necessarily.

The LA-semigroups, after the 1970s, evolved from the study of a diverse generalization of groups and semigroups. It has become a separate branch within itself with a considerable number of research results. The reason for its procession is its natural existence in almost all mathematical contexts in which groups and semigroups have been developed.

The study of LA-semigroups has wide applications in the locally associative LA-semigroups, abelian groups, the theory of fuzzy LA-semigroups, ternary semihypergroups, Γ -semihypergroups, neutrosophic LA-semigroups, soft sets and the theory of non-commutative groupoids [42,43,44,45,46,47,48,49]. Here, we propose a new scheme for the encryption of images based on an inverse LA-semigroup and a modified nonlinear chaotic map, which has better confusion and diffusion characteristics that are necessary for a modern substitution-permutation network.

This article comprises of five sections. In Section 2, we introduce fundamentals of a novel structure inverse LA-semigroups. In Section 3, we propose an algorithm for the encryption of images. The efficiency and safety measures for the suggested algorithm are examined in Section 4. The numerical measures are also discussed in Section 4 to examine the response of suggested scheme against differential attacks. Finally, give conclusions in the Section 5. 

## 2. Preliminaries

This section is primarily related to some standard definitions, which will be quite useful in subsequent sections.

**Definition** **1.** *An LA-semigroup is a pair* (L,∗) *where* A  *is a non-empty set;* ∗:L × L→L *satisfies* (a∗b)∗c=(c∗b)∗a *for all* a,b,c∈L *. One can easily observe that the medial identity* (a∗b)∗(c∗d)=(a∗c)∗(b∗d) *naturally holds in the LA-semigroups. It is important to mention here that every LA-semigroup is always medial but its converse is not true.*

**Definition** **2.** *An LA-semigroup* (L,∗) *satisfying the left permutable law* a∗(b∗c)=b∗(a∗c) is called LA**-semigroup. An LA-semigroup with left identity is always LA**-semigroup and LA**-semigroup naturally satisfies the paramedial identity (a∗b)∗(c∗d)=(d∗b)∗(c∗a) *.*

We provide counter examples of three groupoids of order 5 in Table 2, where (i) is a medial groupoid but not an LA-semigroup, (ii) is a left permutable groupoid, which is also paramedial but not an LA-semigroup, (iii) is a left permutable groupoid with left identity but not an LA-semigroup (see Table 2).

**Definition** **3.** *An LA-semigroup* (L,∗) *equiped with left identity* e*, that is,* e∗a=a *for all* a∈L *is LA-monoid. One can notice that left permutable law and paramedial law always hold in an LA-monoid* L. We also investigate a paramedial groupoid with a left identity is LA**-semigroup. An element a∈L *for which there exist an element* $a^{-1}\in L$ *such that* $a^{-1}*a=e$*, then* $a^{-1}$ *is called left inverse of* a*. Right inverse for an element in an LA-monoid can also be defined analogously. It is easy to observe that if* $a^{-1}\in L$ *is the left inverse for an element* a∈A*, that is,* $a^{-1}*a=e$*. Then* $a*a^{-1}=\left(e*a\right)*a^{-1}=\left(a^{-1}*a\right)*e=e*e=e$*. Then* $a^{\prime }=e*a^{\prime }=\left(a^{-1}*a\right)*a^{\prime }=\left(a^{\prime }*a\right)*a^{-1}=e*a^{-1}=a^{-1}$*. Showing that left and right inverses are unique in an LA-monoid. An LA-monoid, in which each element has its left inverse element is known as LA-group.*

**Definition** **4.** *An LA-semigroup* (L,∗) *satisfying the weak associative law* (a∗b)∗c=b∗(a∗c) is called LA*-semigroup. The identities (a∗b)∗c=b∗(a∗c) and (a∗b)∗c=b∗(c∗a) *are equivalent in an LA-semigroup* L *.*

**Definition** **5.** *LA-group is an LA-monoid in which each element has a unique left multiplicative inverse. LA-monoids are special cases of LA-semigroups whereas LA-groups are special cases of LA-monoid.*

**Definition** **6.** *An LA-semigroup* L *is unipotent if* aa=bb *for all* a,b∈L *. We define a transformation* O(L): L→L *by* $O_{L}\left(a\right)=aa$ *for all* a∈L *.*

**Proposition** **1.** *An LA-semigroup* L *is unipotent if and only if* $ker\left(O_{L}\right)=L\;\times \;L$ *, where* $ker\left(O_{L}\right)=\left\{\left(a,b\right)\in L\;\times \;L:O_{L}\left(a\right)=O_{L}\left(b\right)\right\}$ *.*

**Lemma** **1.** *Every unipotent left cancellative LA-semigroup is paramedial.*

Let (L,∗₁) and (L,∗₂) be any two LA-semigroups of the same order. A mapping φ:(L,∗₁)→(L,∗₂) is a homomorphism if it preserves the multiplication, that is, (a∗₁b)φ=(a)φ∗₂(b)φ and an anti-homomorphism if it reverts the multiplication that is, (a∗₁b)φ=(b)φ∗₂(a)φ. If such mapping is bijective, then it is known as isomorphism and anti-isomorphism respectively. If such a bijection exists, then the groupoids are isomorphic and anti-isomorphic respectively. 

For instance, if two LA-semigroups have isomorphic tables, they have the same structural properties. So we say that an isomorphism is an action between two multiplication tables. If we are provided with a permutation φ of the elements of A, we transform the table by permuting the rows according to φ, then each column, and permuting the values at the end. An anti-isomorphism is an action followed by transposing the resulting table of an isomorphism. The outcome of applying the permutation (b,d) is given in Table 3 (ii). Table 3 (i) and Table 3 (ii) are isomorphic. If A has n elements then atmost multiplication tables isomorphic to given LA-semigroup.

**Theorem** **1.** *Let* L *be an LA-semigroup satisfying paramedical law. Then, one has*

(*i*) $O_{L}$ is an anti-endomorphism.
(*ii*) $O_{L}\left(L\right)$*is sub LA-semigroup of*L.
(*iii*) $ker\left(O_{L}\right)$ is a congruence relation.

**Definition** **7.** *An LA-semigroup* L *, in which for every* u∈L *there exists a unique* v∈L *for which* (uv)u=u *and* (vu)v=v is called an inverse LA-semigroup. This notion was made known by Mushtaq and Iqbal in Reference [9]. They proved some interesting facts and a famous Wagner Preston theorem on the representation of inverse LA-semigroups [9]. An inverse LA*-semigroup and inverse LA**-semigroup are defined analogously. Some interesting results are that every LA-group is an inverse LA-semigroup more precisely an inverse LA**-semigroup but the converse is not true [10]. Presentation of a semigroup is a set of generators and relations which completely depicts a particular semigroup of a finite or an infinite semigroup. Ruskuc worked on semigroup presentations in his PhD thesis [11]. Here, we find that $\Pi =\langle a|a^{257}=a=aa^{128},a^{m}a^{256-m}=a^{256};1\leq m\leq 256>$ represents an inverse LA**-semigroup of order 256 generated by a *. Here, by an S-box of order* 16 *, we mean a latin square of size* 16 × 16 *whose entries are selected from a set of* 256 *different symbols in such a way that there is no repetition in any row and column of the table.*

## 3. Proposed Digital Image Encryption Algorithm

The present section deals with the encryption procedure.

### 3.1. Image Encryption

**Figure 1:**
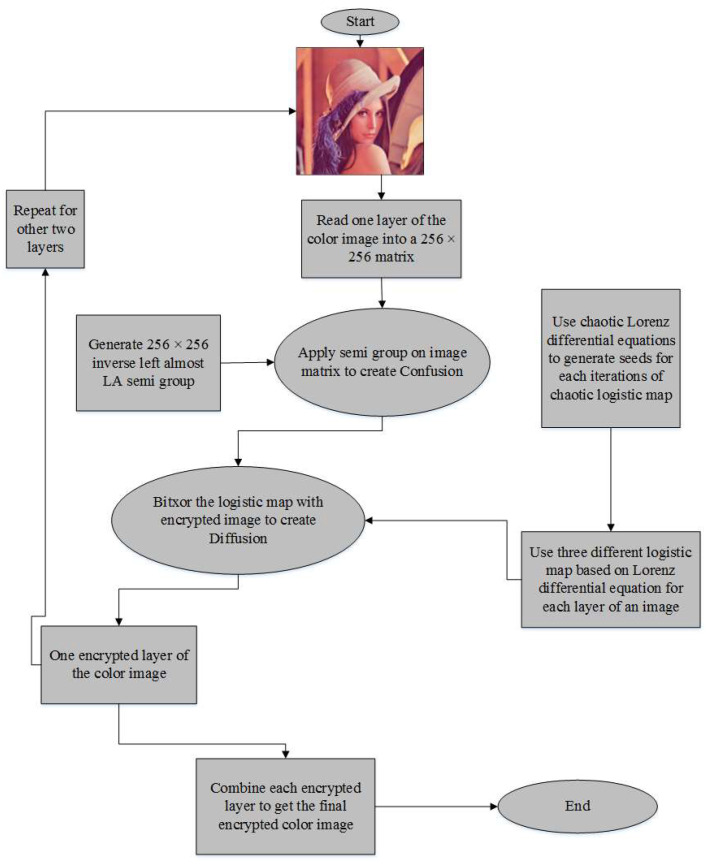
Proposed encryption algorithm.

Designed image encryption techniques comprise of confusion and diffusion. As illustrated in Figure 1, the encryption method is based on the steps given below:

Step 1: Take a standard digital color image of size m × n.

Step 2: Read the inverse left almost LA-semigroup of order m × n.

Step 3: Apply a substitution transformation by using the LA-semigroup as listed in Step 2, which adds confusion to the proposed algorithm.

Step 4: Generate chaotic sequences using Lorenz chaotic differential equations with a logistic map (seed values for each iteration comes from the Lorenz chaotic differential equation utilized three chaotic logistic maps used seeds from x, y and z directions solutions of Lorenz chaotic differential equations).

Step 5: Apply a bitwise addition under modulo 2, of confused image produced in Step 3 with the chaotic sequences generated in Step 4 for each layer of the digital image that uses *x* component values for the red layer, *y* component values from the green layer and *z* component values of the logistic map for the blue layer of a given image. 

Step 6: Apply all of the above steps on each layer of the digital image.

Step 7: Display the encrypted image.

### 3.2. Image Decryption

**Figure 2:**
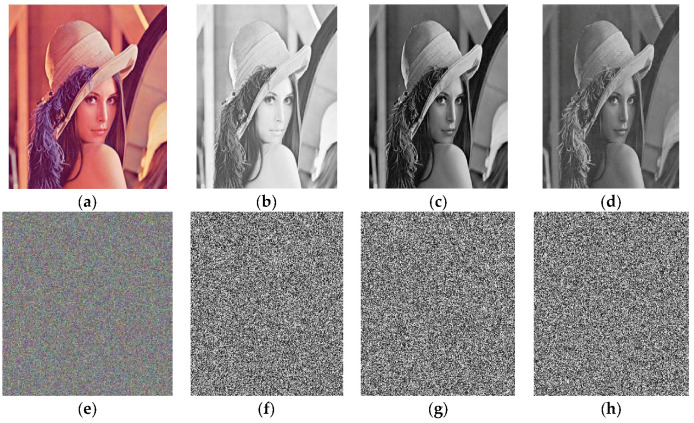
The images (**e**), (**f**), (**g**) and (**h**) are layer-wise encrypted results for images of Lena (**a**) plain image; (**b**) red; (**c**) green and (**d**) blue components respectively.

**Figure 3:**
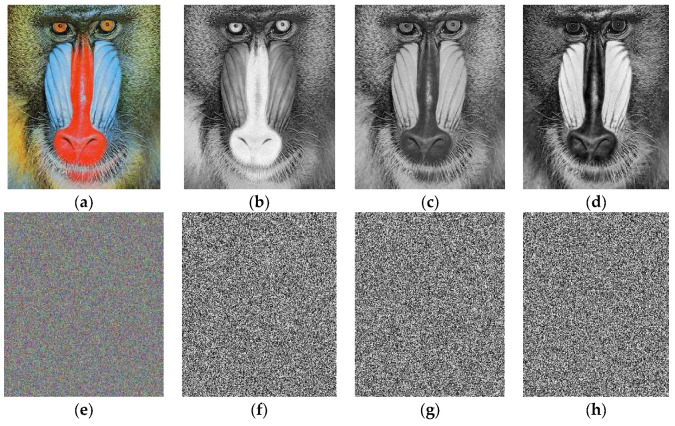
The images (**e**), (**f**), (**g**) and (**h**) are layer-wise encrypted results for images of Baboon (**a**) plain image; (**b**) red; (**c**) green and (**d**) blue components respectively.

**Figure 4:**
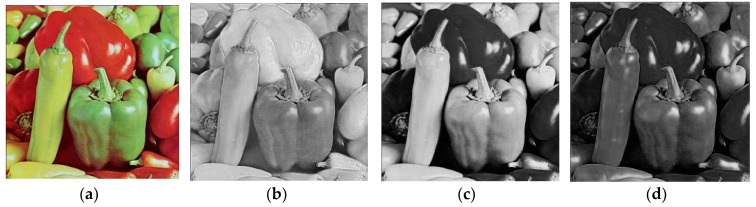

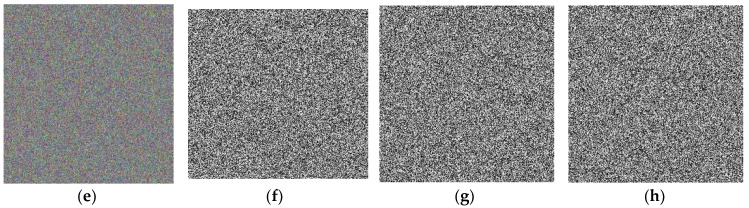
The images (**e**), (**f**), (**g**) and (**h**) are layer-wise encrypted results for images of Peppers (**a**) plain image; (**b**) red; (**c**) green and (**d**) blue components respectively.

This method is used as a reverse process of our encryption procedure. The encrypted images through our proposed algorithm can be seen in Figure 2, Figure 3 and Figure 4 respectively. 

## 4. Security Analysis of the Proposed Algorithm

Here, we apply some statistical measures on the typical digital contents to examine the safety during execution of the proposed encryption scheme. These measurements are strictly based on a precise evaluation, a realistic inspection and an inconsistency criterion for the encryption of images. 

### 4.1. Uniformity Analysis of Image Pixels

**Figure 5:**
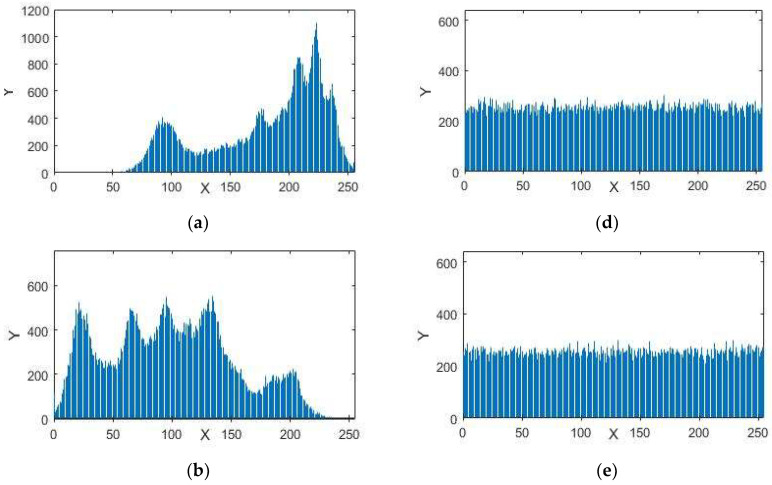

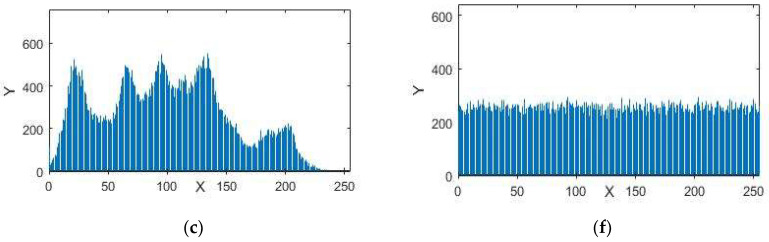
Histograms (**a**) red component; (**b**) green component and (**c**) blue component for the original images of Lena with size 256 × 256. Histograms (**d**) red component; (**e**) green component; (**f**) blue component for the encrypted images of Lena.

**Figure 6:**
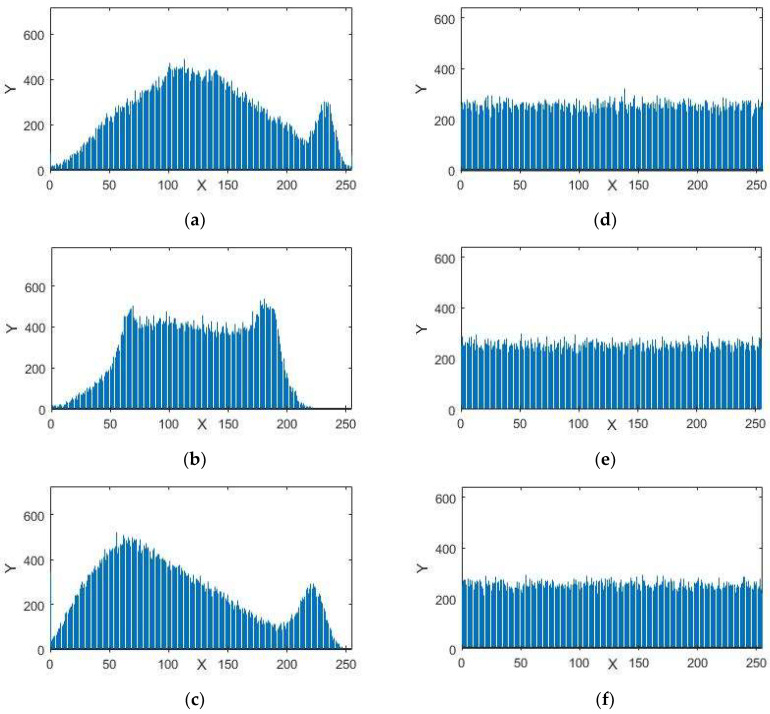
Histograms (**a**) red component; (**b**) green component and (**c**) blue component for the original images of Baboon with size 256 × 256. Histograms (**d**) red component; (**e**) green component; (**f**) blue component for the encrypted images of Baboon.

**Figure 7:**
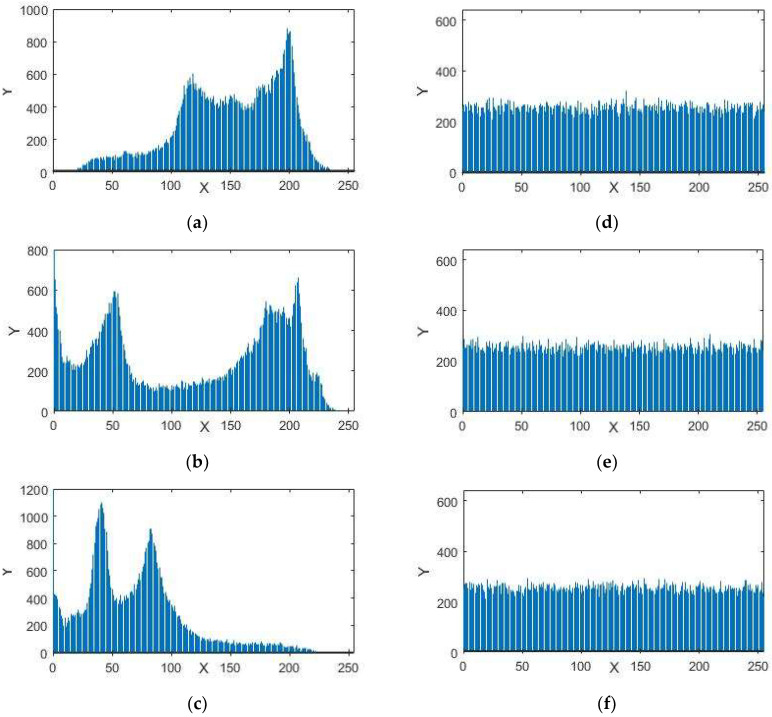
Histograms (**a**) red component; (**b**) green component and (**c**) blue component for the original images of Peppers with size 256 × 256. Histograms (**d**) red component; (**e**) green component; (**f**) blue component for the encrypted images of Peppers.

A histogram of an image provides information about the circulation of the pixel intensity esteems for an image. A protected framework in encryption can have an identical histogram to resist statistical assaults. The histograms in Figure 5, Figure 6 and Figure 7 represent the standard and encrypted images of Lena, Baboon, and Peppers. From Figure 5, Figure 6 and Figure 7, we analyzed that the histograms of the standard images are not uniform, whereas the histograms of the encrypted digital images are uniform. The uniformity of pixel heights in the histograms of the encrypted images creates difficulty for attackers to find the clue for the maximum information region. 

### 4.2 Correlation Analysis for Adjacent Pixels

**Figure 8:**
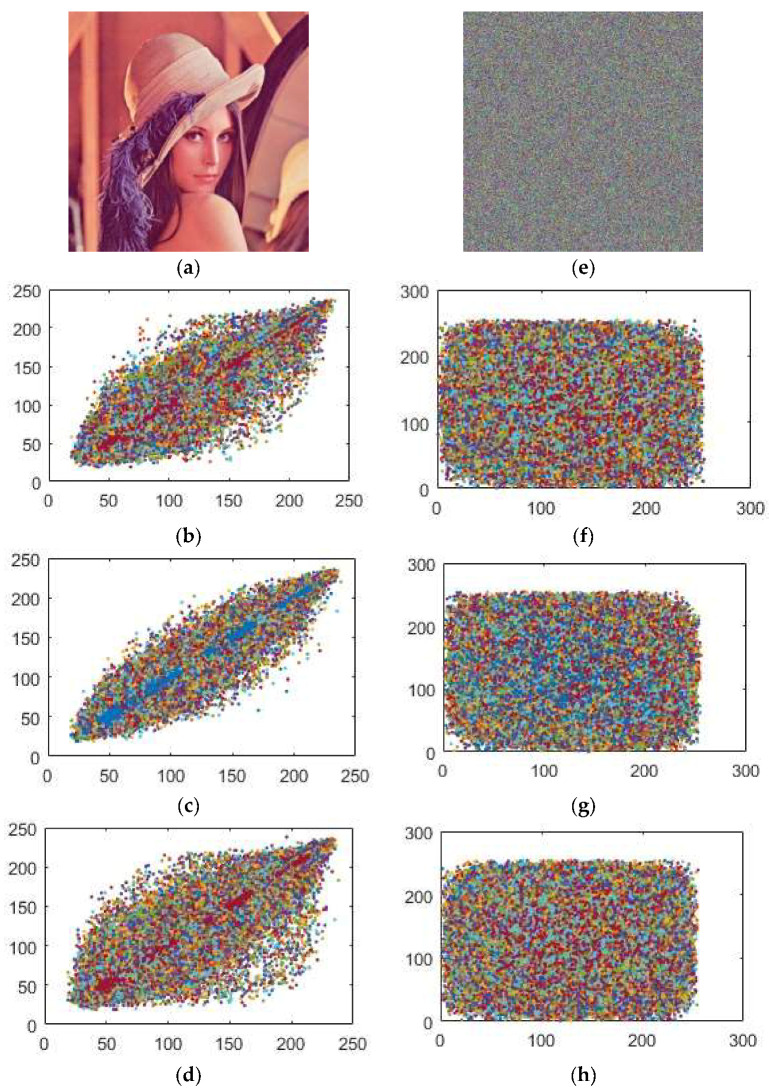
Correlation coefficients between the pairs of the pixels for (**a**) Original Lena image; (**b**) horizontally; (**c**) vertically and (**d**) diagonally (**e**) enciphered Lena image; (**f**) horizontally; (**g**) vertically; (**h**) diagonally.

**Figure 9:**
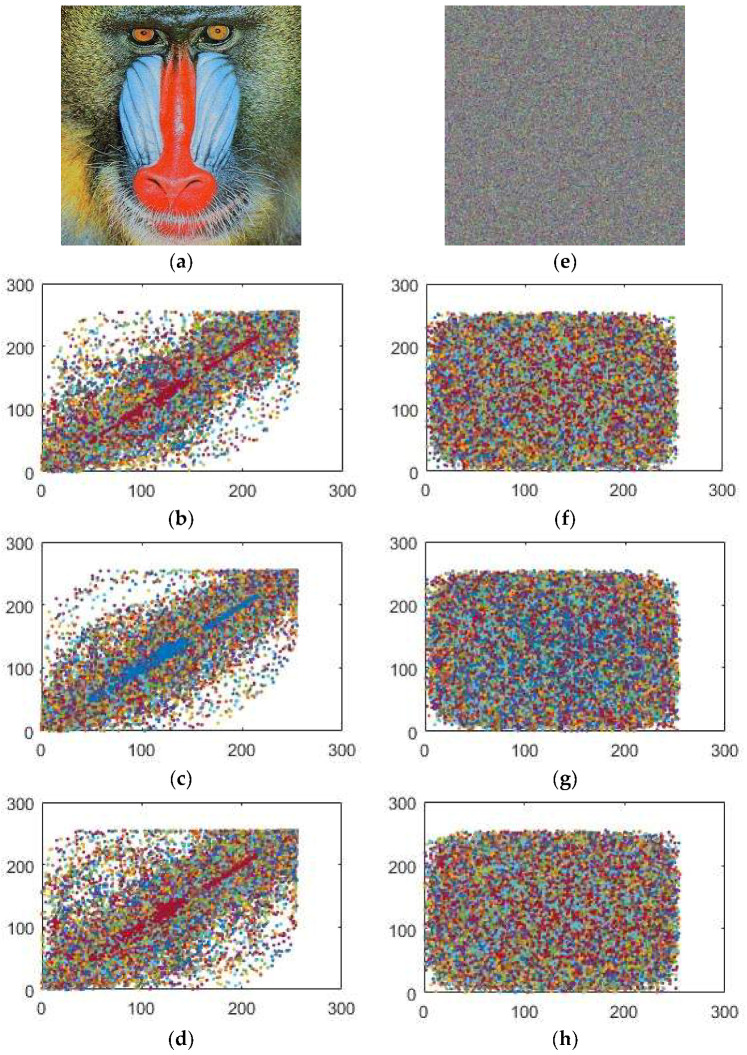
Correlation coefficients between the pairs of the pixels for (**a**) original Baboon image; (**b**) horizontally; (**c**) vertically and (**d**) diagonally (**e**) enciphered Baboon image; (**f**) horizontally; (**g**) vertically; (**h**) diagonally.

**Figure 10:**
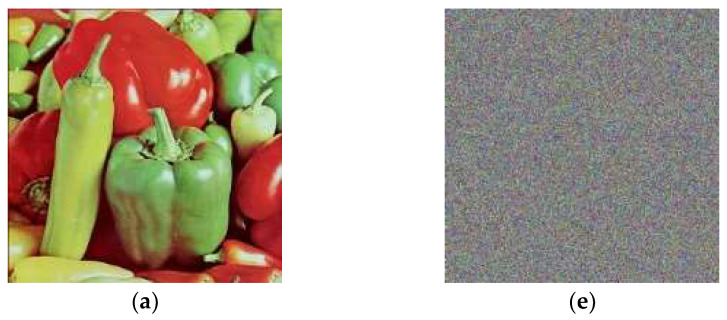

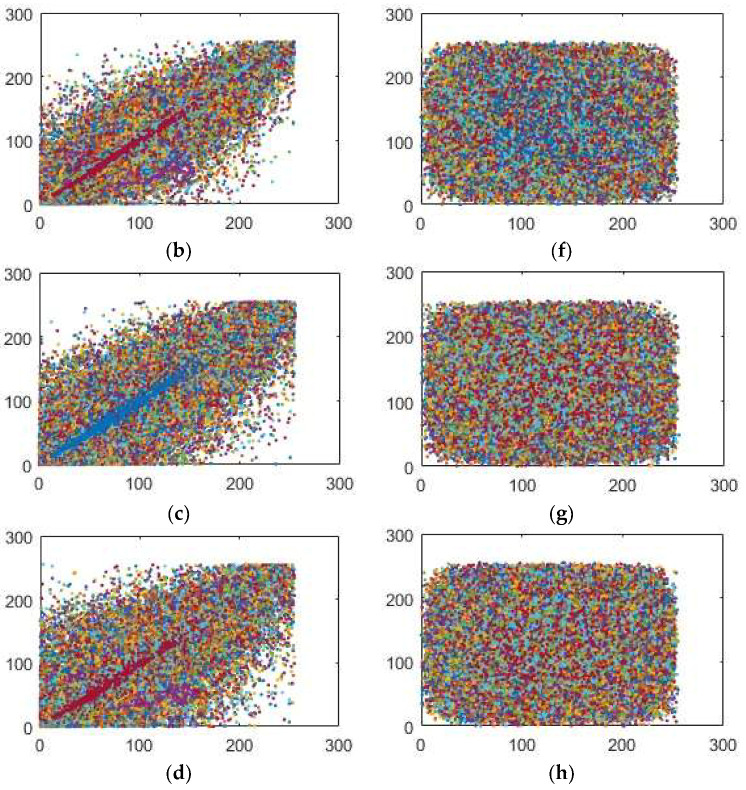
Correlation coefficients between the pairs of the pixels for (**a**) original Peppers image; (**b**) horizontally; (**c**) vertically and (**d**) diagonally (**e**) enciphered Peppers image; (**f**) horizontally; (**g**) vertically; (**h**) diagonally.

The purpose of the correlation analysis was to examine the connections of neighboring pixels in the original and encrypted images. Mathematically, the correlation coefficients $r_{X,Y}$ of two neighboring pixels is defined as:

(1) $$r_{X,Y}=\frac{Cov\left(X,Y\right)}{\sqrt{Var\left(X\right)Var\left(Y\right)}},$$

where *X* and *Y* are the estimations of two neighboring pixels of gray scale image, *Var*(*X*) and *Var*(*Y*) are deviations of *X* and *Y* individually and *Cov*(*X,Y*) represents the covariance. The correlation coefficients of the plain and encrypted digital images have a distinctive substance displayed in Table 4, Table 5, Table 6 and Table 7 identified by the plain and enciphered digital images are provided in Figure 8, Figure 9 and Figure 10. In addition, Table 4 contains the quantified evaluation of the correlation coefficient demonstrating the diffusion of the unique and encoded images horizontally, vertically and diagonally. Presently, we consider 2000 pairs of randomly selected neighboring pixels to look over the original and the enciphered images horizontally, vertically and diagonally. In Table 4, the correlation coefficients for the green, blue and red parts of the encrypted images are quite small, which implies an irrelevant correlation between adjoining pixels.

In addition, high correlation coefficients for the red, green and blue parts of the original images make data spillage conceivable. Table 6 provides us with similar position correlations for the red, green and blue parts, while Table 7 gives the adjoining position correlations for the red, green and blue parts. From Table 6 and Table 7, we analyze that the correlation coefficients of the encrypted digital images for the red, green and blue parts are all lower than −0.002, while the greatest correlation coefficient for the original images is 0.9652 in the event of the Lena image, 0.8310 for the Baboon and 0.9444 for the Peppers image, which indicates that the correlations for the red, green and blue parts of the encrypted images are adequately diminished. Therefore, our encryption scheme is highly defensive against statistical attacks.

In addition, we plotted the correlation coefficients for the red, green and blue parts of the original images in Figure 8a–d, Figure 9a–d and Figure 10a–d and the encrypted images Figure 8e–h, Figure 9e–h and Figure 10e–h toward every directions, as delineated in Figure 8, Figure 9 and Figure 10. The solid correlation between adjoining pixels of the plain images is apparent as the specks are congregated along the slanting in Figure 8a–d, Figure 9a–d and Figure 10a–d. Nonetheless, the specks are scattered over the whole plane in Figure 8a–d, Figure 9a–d and Figure 10a–d, which shows that the correlation is incredibly diminished in the encrypted digital images.

### 4.3. Pixel Modification Based Measurements

The quality of an image depends upon the pixel difference which is calculated by means the mean square error (MSE), average difference (AD), maximum difference (MD), normalized absolute error (NAE), normalized cross correlation (NCC), structure content (SC) and peak signal to noise ratio) values (PSNR). These metrics are used for the comparison of unlike images.

#### 4.3.1. Mean Square Error (MSE)

An encrypted image should not be equivalent to the original digital image due to the application of the encryption scheme over the plain image, which surely adds some noise to the actual digital content. We find MSE of the plain and encrypted images to analyze the level of enciphering. Mathematically, MSE is defined as:

(2) $$MSE=\frac{\sum_{j=1}^{m}\sum_{k=1}^{n}{\left(P_{jk}-C_{jk}\right)}^{2}}{m\;\times \;n}$$

where $P_{ij}$ and $C_{ij}$ are the pixels positioned in the *j*-th row and *k*-th column of the plain and enciphered images respectively. A larger value of the MSE enhances the security of the encryption scheme.

#### 4.3.2. Peak Signal to Noise Ratio (PSNR)

Mathematically, PSNR is defined as:

(3) $$\mathrm{PSNR}=20\log_{10}\left[\frac{I_{MAX}}{\sqrt{MSE}}\right],$$

where $I_{MAX}$ is the maximum value of pixel which can occur. The low value of PSNR shows the more difference of the original and enciphered images. In Table 8, we discuss values MSE and PSNR to ensure the versatility of the suggested scheme.

#### 4.3.3. Normalized Absolute Error (NAE)

Mathematically, normalized absolute error (abbreviated as NAE) is defined as:

(4) $$NAE=\frac{\sum_{j=1}^{m}\sum_{k=1}^{n}\left|P_{jk}-C_{jk}\right|}{\sum_{j=1}^{m}\sum_{k=1}^{n}\left|C_{jk}\right|}.$$

It is the proportion of the encrypted digital content to the original image. A bigger estimation of NAE demonstrates the great nature of coming about the scrambled image after the encryption process.

#### 4.3.4. Maximum Difference (MD)

Mathematically, the maximum difference is defined as:

$$MD=Max\left|P_{jk}-C_{jk}\right|,$$

where

(5) j=1,2,…,m, k=1,2,…,n.

It measures the maximum of the error signal. A higher value of the maximum difference indicates that the quality of the encryption scheme is better. 

#### 4.3.5. Average Difference (AD)

The average difference measures the pixel contrast between the original image and its corresponding enciphered image. This quantitative measure is only utilized in object revealing and pattern recognition applications and it can likewise be pertinent to any image preparing applications where we locate the normal distinction between two digital contents. A larger estimation of the AD indicates the great quality of the digital image encryption (see Table 9). Mathematically, the average difference is defined as:

(6) $$AD=\frac{\sum_{j=1}^{m}\sum_{K=1}^{n}\left(P_{jk}-C_{jk}\right)}{m\;\times \;n}.$$

The value of AD is ideally zero for two same digital images.

### 4.4. Similarities Measures

The likenesses between two signals can be estimated through a cross-correlation, structure similarity, and structure content. These are the standard devices for assessing how much two signs are comparable or divergent. It is a basic way to match two image patches, for highlight recognition and in addition a part of more refined systems. The method has a few favorable circumstances. We have used a standardized correlation and structure content with the end goal to demonstrate the dissimilarities among the original and scrambled images.

#### 4.4.1. Normalized Cross Correlation (NCC)

A normalized cross-correlation (NCC) has been normally utilized as a metric to assess the level of likeness (or difference) between two digital images. The normalized cross-correlation is limited in the range between −1 and 1. The setting of the location edge esteem is significantly less difficult than the cross-correlation. The NCC measures the cozy connection between two images, it might be plain and enciphered digital images. All of the correlation-based measures are considered as 1, as the distinction between the two images is considered as zero. In each image, the similitude estimation is done dependent on the direct splendor and complexity varieties of utilizing cross-correlation. Mathematically, normalized cross-correlation is defined as:

(7) $$NCC=\frac{\sum_{j=1}^{m}\sum_{k=1}^{n}P_{jk}\;\times \;C_{jk}}{\sum_{k=1}^{n}{\left(P_{jk}\right)}^{2}}$$

where *m × n* is the size of both plain image *P* and cipher image *C*. The estimation of NCC for encryption varies from or not near unity, which unmistakably means that the proposed scheme includes solid dissimilarities among the pixels of plain and scrambled images (see Table 9).

#### 4.4.2. Structural Content (SC)

This measure viably thinks about the aggregate weight of an original signal to that of a coded or given. It is, hence, a worldwide metric. This measure is likewise called as structural content (SC), and in the event that it is spread at 1, at that point the changed over image is of better quality and a huge estimation of SC implies that the image is of low quality. Mathematically, structural content is defined as:

(8) $$SC=\frac{\sum_{j=1}^{M}\sum_{k=1}^{N}{\left(P\left(j,k\right)\right)}^{2}}{\sum_{j=1}^{M}\sum_{k=1}^{N}{\left(C\left(j,k\right)\right)}^{2}}\;.$$

On account of the plain and encoded images, the estimation of SC is not close to unity because the encryption scheme includes confusion and diffusion-like noise and commotion in the original image. The estimation of SC isn’t near one if there should be an occurrence of all advanced standard shading images (red, green and blue layers) (see Table 9).

### 4.5. Entropy Investigation

Entropy is evaluated to analyze the spreading of the gray scale estimations of the images. The coarser the image is, the bigger the entropy is. For an irregular image with 256 gray levels, the entropy ought to, in a perfect world, be 8 [9]. On the off chance that the entropy of the encrypted image is under 8, there is a probability of consistency, and this is a risk to the anticipated security. Mathematically, we can represent the entropy *H* for a data source y is characterized as:

(9) $$H=-\sum_{i=0}^{2^{N}-\;1}p(y_{i})\log_{2}p(y_{i}),$$

where $2^{N}$ are all possible states of information and *y_i_* is the source images. For a completely sporadic source exuding signs, entropy should be *N*. In Table 10, the entropies of different plain and enciphered image entropies are given as demonstrated by the plain images in Figure 2, Figure 3 and Figure 4. These values are very close to the theoretical value which is 8. Consequently, information spillage in our encryption is negligible and well secured for physical attacks. We have looked at data entropy for our proposed encryption method with the already developed encryption plans. Table 11 shows that the entropy of the offered scheme for the scrambled images are better than the already available algorithms.

In Table 11 and Table 12, we compare the entropy of the proposed algorithm to the already defined algorithms. Our entropies are approximately equal to 8, which is the most suitable value. This minimizes the chance of data spillage during the encryption. Consequently, the proposed image cryptosystem is secure against an entropy assault. In addition, the entropy estimations of the introduced scheme are better than the encryption schemes [3,4,5,6].

### 4.6. Robustness against Differential Attack

Diffusion is a basic parameter to examine the randomness in the encryption scheme. It is an important measurement in the proposed digital image encryption algorithm because it reflects even a minor change in the plain image [4,5]. By and large, the enemy may roll out by a slight improvement. For instance, by altering a single pixel of the original image and after that observe the difference in the outcome. Along these lines, we may discover a significant connection of the original and encrypted image. Since a little change in the original image can create a critical change in the encrypted image. Thus, differential attacks would be exceptionally wasteful and for all intents and purposes futile. On the off chance that an encryption scheme has a decent trademark, the connection between the original image and the encrypted image is extremely complex and it cannot be expected easily. We can measure the diffusion of an encryption scheme by changing a pixel in the original image [6]. To measure the effect of a one-pixel change in the original image and encrypted image, we used three estimations MAE, NPCR and UACI to examine security in the encrypted image against differential attacks. Suppose $C_{1}$ and $C_{2}$ are encrypted images before and after one pixel change in the plain image respectively. Then the MAE, NPCR and UACI are defined as:

(10) $$MAE=\frac{\sum_{j,k}\left|C_{1}\left(j,\;k\right)-C_{2}\left(j,\;k\right)\right|}{m\;\times \;n}$$

(11) $$NPCR=\frac{\sum_{j,k}D\left(j,k\right)}{W\;\times \;H}\;\times \;100\%$$

where

(12) $$D\left(j,k\right)=\{\begin{array}{cc} 0 & C_{1}\left(j,\;k\right)=C_{2}\left(j,\;k\right)\\ 1 & C_{1}\left(j,\;k\right)\neq C_{2}\left(j,\;k\right) \end{array}$$

$C_{1}\left(i,j\right),\;C_{2}\left(i,j\right)$ represent the value of pixel at grid (i,j) and *D*(*i*, *j*) is a bipolar array at grid (i,j).

(13) $$UACI=\frac{1}{W\;\times \;H}\sum_{j=1}^{m}\sum_{k=1}^{n}\left|\frac{C_{1}\left(j,\;k\right)-C_{2}\left(j,\;k\right)}{255}\right|\;\times \;100\%.$$

In Table 13 and Table 14, we have MAE (> 75), (NPCR (> 99%) and UACI (≈ 33%) for the red, green and blue part of the encrypted standard images. These values of MAE, NPCR and UACI are very close to the best-approximated values already available in the literature. Hence, the suggested encryption algorithm can create more difficulty for differential attackers. 

A comparison of the differential analysis with some of the already existing results is listed in Table 15 and Table 16 respectively. 

We have also calculated the pixels difference base and similarities measurements between the original encrypted image $C_{1}$ and the one bit change encrypted image $C_{2}$. The mathematical expressions for the pixels difference and similarity measures for $C_{1}$ and $C_{2}$ are given below:

(14) $$MSE=\frac{\sum_{j=1}^{m}\sum_{K=1}^{n}{\left(C_{1}\left(j,\;k\right)-C_{2}\left(j,\;k\right)\right)}^{2}}{m\;\times \;n}$$

(15) $$PSNR=20\log_{10}\left[\frac{I_{max}}{\sqrt{\frac{\sum_{j=1}^{m}\sum_{K=1}^{n}{\left(C_{1}\left(j,\;k\right)-C_{2}\left(j,\;k\right)\right)}^{2}}{m\;\times \;n}}}\right]$$

(16) $$NAE=\frac{\sum_{j=1}^{m}\sum_{K=1}^{n}\left|C_{1}\left(j,\;k\right)-C_{2}\left(j,\;k\right)\right|}{\sum_{j=1}^{m}\sum_{K=1}^{n}\left|C_{1}\left(j,\;k\right)\right|}$$

$$MD=Max\left|C_{1}\left(j,\;k\right)-C_{2}\left(j,\;k\right)\right|,$$

where

(17) j=1,2,…,m, k=1,2,…,n

(18) $$AD=\frac{\sum_{j=1}^{m}\sum_{K=1}^{n}\left(C_{1}\left(j,\;k\right)-C_{2}\left(j,\;k\right)\right)}{m\;\times \;n}$$

(19) $$NCC=\frac{\sum_{j=1}^{m}\sum_{k=1}^{n}C_{1}\left(j,\;k\right)\;\times \;C_{2}\left(j,\;k\right)}{\sum_{k=1}^{n}{\left(C_{1}\left(j,k\right)\right)}^{2}}$$

(20) $$SC=\frac{\sum_{j=1}^{m}\sum_{k=1}^{n}{\left(C_{1}\left(j,k\right)\right)}^{2}}{\sum_{j=1}^{m}\sum_{k=1}^{n}{\left(C_{2}\left(j,k\right)\right)}^{2}}.$$

The analyses of the pixel difference and similarity measures are shown in Table 17. This shows that a slight difference in the neighboring pixels creates an avalanche effect, which is one of the basic requirements while designing any image encryption technique. The numerical values of MSE, PSNR, NCC, NAE AD, SC and MD for original encrypted image $C_{1}$ and the one bit change encrypted image $C_{2}$ clearly reflect that our proposed algorithm is resistive against differential attacks.

## 5. Conclusions

The present research article provides a new idea for the construction of an image encryption technique. A completely new inverse LA-semi group was investigating and applied for substitution, which is one of the most important components in symmetric encryption. This new mechanism added confusion, which is fundamentally responsible for breaking the pattern between the original and encrypted information. Moreover, we have utilized chaotic continuous systems in order to add diffusion into our proposed image encryption scheme. The proposed idea adds a new milestone for oncoming researchers. 

## Figures and Tables

**Table 1:** Locally associative left almost semigroup (LA-semigroup).

*	0	1	2
0	2	2	1
1	1	1	1
2	1	1	1

**Table 2:** Counter Examples.

(i)						(ii)						(iii)					
*_1_	0	1	2	3	4	*_2_	0	1	2	3	4	*_3_	0	1	2	3	4
0	4	4	4	2	2	0	0	0	0	0	0	0	3	0	4	4	0
1	4	0	0	4	4	1	0	0	0	2	1	1	4	2	0	0	3
2	4	0	4	2	4	2	0	0	0	1	1	2	0	1	2	3	4
3	0	2	2	0	0	3	0	0	0	2	0	3	0	4	3	3	4
4	4	4	4	4	4	4	0	0	0	1	0	4	4	3	0	0	3

**Table 3:** A Multiplication table is mapped to another multiplication under permutation (*b*, *d*).

(i)							(ii)							(iii)					
	*a*	*B*	*c*	*d*	*e*	$\vec{apply\;\left(b,\;d\right)}$		*a*	*d*	*c*	*b*	*e*	$\vec{rearrange}$		*a*	*b*	*c*	*d*	*e*
*a*	*b*	*E*	*e*	*a*	*a*		*a*	*d*	*e*	*e*	*a*	*a*		*a*	*d*	*a*	*e*	*e*	*a*
*b*	*a*	*B*	*b*	*e*	*e*		*d*	*a*	*d*	*d*	*e*	*e*		*b*	*e*	*e*	*a*	*d*	*d*
*c*	*a*	*b*	*c*	*d*	*e*		*c*	*a*	*d*	*c*	*b*	*e*		*c*	*a*	*b*	*c*	*d*	*e*
*d*	*e*	*A*	*a*	*c*	*b*		*b*	*e*	*a*	*a*	*c*	*d*		*d*	*a*	*c*	*d*	*a*	*e*
*e*	*e*	*a*	*a*	*b*	*b*		*e*	*e*	*a*	*a*	*d*	*d*		*e*	*e*	*d*	*a*	*a*	*d*

**Table 4:** Color components-wise correlation coefficient of cipher images.

Image	Layer	Correlation of Encrypted Image	Correlation of Altered Encrypted Image
Lena	Red	−0.019408	−0.026560
	Green	0.005199	0.016749
	Blue	−0.057938	−0.013504
Baboon	Red	−0.017262	−0.062436
	Green	−0.018564	−0.039240
	Blue	−0.011867	0.037551
Airplane	Red	−0.035036	0.025052
	Green	0.040117	−0.065009
	Blue	−0.015017	0.014972
Pepper	Red	−0.002283	−0.035236
	Green	−0.019859	0.007575
	Blue	−0.028659	0.044896
House	Red	−0.034227	0.037809
	Green	−0.079819	−0.001682
	Blue	0.020816	−0.014746
Jelly beans	Red	−0.069787	−0.002181
	Green	−0.029373	0.018362
	Blue	−0.003245	−0.027437
Tree	Red	0.015815	0.008496
	Green	0.032206	0.007798
	Blue	−0.021771	−0.015972
Splash	Red	−0.030921	−0.023138
	Green	0.072383	0.012479
	Blue	0.005316	−0.024963
Sail boat on lake	Red	0.035408	0.000482
	Green	−0.004099	−0.030648
	Blue	0.020935	0.020618

**Table 5:** Correlation coefficients of original and encrypted images.

Standard Images	Original Image			Encrypted Image		
	Horizontal	Vertical	Diagonal	Horizontal	Vertical	Diagonal
Lena	0.9339	0.9652	0.9076	−0.0043	−0.0090	−0.0031
Baboon	0.8310	0.7737	0.7723	−0.0029	−0.0079	0.0026
Peppers	0.9392	0.9003	0.9444	−0.0028	−0.0090	−0.0007

**Table 6:** Correlation coefficients of the plain and cipher image for the Lena color image of size 256 × 256.

Standard Images	Plain Image			Encrypted Image		
	Red	Green	Blue	Red	Green	Blue
Horizontal	0.9339	0.9044	0.8609	−0.0084	−0.0028	−0.0072
Vertical	0.9652	0.9464	0.9086	−0.0052	−0.0066	−0.0098
Diagonal	0.9076	0.8796	0.8371	−0.0016	0.0012	0.0013

**Table 7:** Comparison between the correlation coefficients of the proposed scheme and recent techniques using Lena image.

	Correlation Directions		
	Horizontal	Vertical	Diagonal
Proposed encryption scheme	−0.0043	−0.0090	−0.00310
Reference [3]	0.06810	0.08450	-
Reference [15]	0.21570	0.05810	0.05040
Reference [17]	0.00720	0.00580	0.00310
Reference [18]	0.02140	0.04650	−0.0090
Zhang et al. [26]	0.08200	0.04000	0.00500
Zhou et al. [27]	0.01200	0.02700	0.00700
Etimadi et al. [30]	0.00500	0.01100	0.02300

**Table 8:** MSE and PSNR of the suggested scheme.

Images	Pixel Difference Based Measures	
	MSE	PSNR
Lena	4859.03	11.30
Baboon	6399.05	10.10
Peppers	7274.44	9.55

**Table 9:** Pixel difference-based and correlation-based measures of the proposed encryption scheme.

		Pixel Difference Measures					Correlation Measures	
Image	Layer	MSE	PSNR	AD	MD	NAE	NCC	SC
Lena	Red	10637	7.8625	52.3109	255	0.4674	0.6598	1.6004
	Green	9245.2	8.4716	−28.9211	235	0.7968	0.9983	0.5788
	Blue	7169.4	9.5760	−22.2776	229	0.6713	1.0952	0.5632
Baboon	Red	8740.1	8.7156	1.9610	255	0.5938	0.8259	0.9174
	Green	7802.8	9.2083	−5.9805	230	0.6025	0.9106	0.7810
	Blue	9714.3	8.2567	−21.8818	244	0.7670	0.9038	0.6819
Airplane	Red	10039	8.1138	49.6505	255	0.4616	0.6758	1.5368
	Green	10662	7.8524	49.5975	242	0.4750	0.6641	1.5619
	Blue	10764	7.8110	63.1120	255	0.4458	0.6460	1.7249
Pepper	Red	8041.9	9.0772	22.1327	234	0.4941	0.7808	1.1235
	Green	10993	7.7197	−11.5843	242	0.7396	0.7842	0.8678
	Blue	11048	7.6980	−60.3558	220	1.2788	1.3319	0.2955

**Table 10:** Entropies of various plain and enciphered images.

Image	Layer	Plain Image	Altered Image	Enciphered Image	Enciphered Altered Image
Lena	Red	7.2352	7.2353	7.9965	7.9975
	Green	7.5812	7.5814	7.9970	7.9970
	Blue	7.5682	7.5683	7.9971	7.9971
Baboon	Red	7.7766	7.7766	7.9965	7.9967
	Green	7.4911	7.4911	7.9968	7.9973
	Blue	7.7546	7.7546	7.9973	7.9973
Airplane	Red	6.8505	6.8505	7.9972	7.9972
	Green	6.8622	6.8622	7.9969	7.9968
	Blue	6.4537	6.4537	7.9972	7.9972
Pepper	Red	7.3843	7.3843	7.9976	7.9970
	Green	7.6230	7.6230	7.9971	7.9971
	Blue	7.1437	7.1438	7.9971	7.9971
House	Red	6.4310	6.4310	7.9970	7.9974
	Green	6.2320	6.2321	7.9969	7.9971
	Blue	6.5389	6.5389	7.9966	7.9970
Jelly beans	Red	5.2626	5.2626	7.9968	7.9968
	Green	6.5464	6.5463	7.9971	7.9973
	Blue	5.6947	5.6947	7.9971	7.9972
Tree	Red	7.2104	7.2104	7.9967	7.9966
	Green	6.9207	6.9206	7.9973	7.9973
	Blue	7.4136	7.4136	7.9973	7.9969
Splash	Red	7.2022	7.2022	7.9971	7.9973
	Green	7.0099	7.0100	7.9972	7.9970
	Blue	6.3056	6.3056	7.9971	7.9972

**Table 11:** Comparison between the entropies for 256 × 256 Lena image.

Algorithm	Entropy
Proposed	7.9968
Sun’s algorithm [31]	7.9965
Baptista’s algorithm [31]	7.9260
Wong’s algorithm [31]	7.9690
Xiang’s algorithm [31]	7.9950

**Table 12:** Comparison of the entropy of the proposed algorithm to the already defined algorithms.

Encryption Techniques	Test Image	Color Components of Original Image			Color Components of Encrypted Image		
		Red	Green	Blue	Red	Green	Blue
Proposed scheme	Lena	7.2933	7.5812	7.5682	7.9965	7.9970	7.9971
Reference [32]	Lena	7.2933	7.5812	7.5682	7.9903	7.9890	7.9893
Reference [5]	Lena	7.2933	7.5812	7.5682	7.9732	7.9750	7.9715
Reference [4]	Lena	7.2933	7.5812	7.5682	7.9791	7.9802	7.9827
Reference [5]	Lena	7.2933	7.5812	7.5682	7.9871	7.9881	7.9878
Reference [4]	Lena	7.2933	7.5812	7.5682	7.9874	7.7872	7.7866
Reference [6]	Lena	7.2933	7.5812	7.5682	7.9278	7.9744	7.9705

**Table 13:** The evaluation of the security measurements of the suggested encrypted algorithm.

Test Images	NPCR			UACI			MAE
	Max	Min	Mean	Max	Min	Mean	
Lena	99.997	99.612	99.713	34.43	33.21	33.87	79.22
Fruits	99.994	99.515	99.698	33.98	33.02	33.71	83.45
Parrot	99.998	99.597	99.869	33.53	33.11	33.24	75.38

**Table 14:** The evaluation of the security measurements of the suggested encrypted algorithm.

Image	Layer	NPCR	UACI
Lena	Red	0.99592	0.33497
	Green	0.99595	0.33325
	Blue	0.99600	0.33223
Baboon	Red	0.99594	0.33589
	Green	0.99563	0.33352
	Blue	0.99623	0.33408
Airplane	Red	0.99624	0.33469
	Green	0.99617	0.33473
	Blue	0.99591	0.33625
Pepper	Red	0.99577	0.33446
	Green	0.99594	0.33453
	Blue	0.99607	0.33747
House	Red	0.99551	0.33487
	Green	0.99609	0.33492
	Blue	0.99603	0.33284
Jelly beans	Red	0.99612	0.33317
	Green	0.99617	0.33408
	Blue	0.99636	0.33406
Tree	Red	0.99627	0.33385
	Green	0.99620	0.33518
	Blue	0.99632	0.33577
Splash	Red	0.99606	0.33522
	Green	0.99600	0.33504
	Blue	0.99601	0.33395
Sail boat on lake	Red	0.99618	0.33718
	Green	0.99604	0.33429
	Blue	0.99609	0.33493

**Table 15:** A comparison of calculated UACI and NPCR for 512 × 512 plain Lena image.

Standard Lena Image	Suggested	Ref. [19]	Ref. [20]	Ref. [21]	Ref. [22]	Ref. [23]	Ref. [24]
UACI	0.3392	0.3362	0.3351	0.3360	0.3351	0.3351	0.3342
NPCR	0.9973	0.9961	0.9961	0.9963	0.9961	0.9960	0.9967

**Table 16:** NPCR, UACI values for the color components of the digital Lena image of size 256 × 256.

Channel	Metrics	Lena	Ref. [33]	Ref. [34]
Red	NPCR	0.99592	0.996013	0.996108
	UACI	0.33497	0.334210	0.334525
Green	NPCR	0.99595	0.996131	99.60580
	UACI	0.33325	0.334485	0.334798
Blue	NPCR	0.99600	0.996226	0.996057
	UACI	0.33223	0.334815	0.334387

**Table 17:** Pixel difference analysis for the one bit change encrypted image with the plain image.

Image	Layer	MSE	PSNR	NCC	AD	SC	MD	NAE
Lena	Red	10770	7.8088	0.6580	52.5558	1.5999	255	0.4713
	Green	9179.8	8.5025	0.9924	−28.0862	0.5846	247	0.7937
	Blue	7168.7	9.5764	1.0933	−22.1300	0.5645	226	0.6720
Baboon	Red	8683.7	8.7437	0.8251	2.2474	0.9212	255	0.5919
	Green	7763.9	9.2300	0.9082	−5.6691	0.7854	225	0.6008
	Blue	9679.3	8.2723	0.9107	−22.4863	0.6766	249	0.7655
Airplane	Red	10116	8.0807	0.6718	50.4712	1.5505	253	0.4635
	Green	10626	7.8670	0.6634	49.7774	1.5679	248	0.4740
	Blue	10648	7.8583	0.6491	62.6407	1.7159	246	0.4427
Pepper	Red	8043.9	9.0761	0.7821	21.8081	1.1200	255	0.4943
	Green	11139	7.6624	0.7790	−11.5038	0.8699	246	0.7473
	Blue	11127	7.6670	1.3306	−60.3176	0.2947	228	1.2846

## References

[1] Xie E.Y., Li C., Yu S., Lü J. (2017). On the cryptanalysis of Fridrich’s chaotic image encryption scheme. Signal Process..

[2] Li C., Lin D., Lü J. (2017). Cryptanalyzing an Image-Scrambling Encryption Algorithm of Pixel Bits. IEEE MultiMedia.

[3] Rhouma R., Meherzi S., Belghith S. (2009). OCML-based colour image encryption. Chaos Soliton. Fractals.

[4] Liu H., Wang X. (2011). Color image encryption using spatial bit level permutation and high-dimension chaotic system. Opt. Commun..

[5] Liu H., Wang X., Kadir A. (2013). Color image encryption using Choquet fuzzy integral and hyper chaotic system. Optik.

[6] Kadir A., Hamdulla A., Guo W. (2014). Color image encryption using skew tent map and hyper chaotic system of 6th-order CNN. Optik.

[7] Waseem H.M., Khan M. (2018). Information Confidentiality Using Quantum Spinning, Rotation and Finite State Machine. Int. J. Theor. Phys..

[8] Khan M., Shah T., Batool S.I. (2017). A new approach for image encryption and watermarking based on substitution box over the classes of chain rings. Multimed. Tools Appl. November.

[9] Belazi A., Khan M., El-Latif A.A.A., Belghith S. (2017). Efficient cryptosystem approaches: S-boxes and permutation substitution-based encryption. Nonlinear Dyn..

[10] Khan M., Asghar Z. (2018). A novel construction of substitution box for image encryption applications with Gingerbreadman chaotic map and S8 permutation. Neural Comput. Appl..

[11] Khan M., Shah T. (2016). Construction and applications of chaotic S-boxes in image encryption. Neural Comput. Appl..

[12] Khan M., Shah T. (2015). An efficient construction of substitution box with fractional chaotic system. Signal Image Video Process..

[13] Khan M., Shah T. (2015). An efficient chaotic image encryption scheme. Neural Comput. Appl..

[14] Khan M., Shah T. (2015). A novel construction of substitution box with Zaslavskii chaotic map and symmetric group. J. Intell. Fuzzy Syst..

[15] Huang C.K., Nien H.H. (2009). Multi chaotic systems based pixel shuffle for image encryption. Opt. Commun..

[16] Mondal B., Kumar P., Singh S. (2018). A chaotic permutation and diffusion based image encryption algorithm for secure communications. Multimed. Tools Appl..

[17] Enayatifar R., Abdullah A.H., Isnin I.F. (2014). Chaos-based image encryption using a hybrid genetic algorithm and a DNA sequence. Opt. Lasers Eng..

[18] Zhen P., Zhao G., Min L., Jin X. (2016). Chaos-based image encryption scheme combining DNA coding and entropy. Multimed. Tools Appl..

[19] Khan M. (2015). A novel image encryption scheme based on multiple chaotic S-boxes. Nonlinear Dyn..

[20] Yang B., Liao X. (2018). A new color image encryption scheme based on logistic map over the finite field *Z_N_*. Multimed. Tools Appl..

[21] Enayatifar R., Abdullah A.H., Isnin I.F., Altameem A., Lee M. (2017). Image encryption using a synchronous permutation-diffusion technique. Opt. Lasers Eng..

[22] Hamza R., Titouna F. (2016). A novel sensitive image encryption algorithm based on the Zaslavsky chaotic map. Inf. Secur. J. Glob. Perspect..

[23] Tong X.J., Zhang M., Wang Z., Ma J. (2016). A joint color image encryption and compression scheme based on hyperchaotic system. Nonlinear Dyn..

[24] Zhang Y.S., Xiao D. (2014). Self-adaptive permutation and combined global diffusion for chaotic color image encryption. AEU Int. J. Electron. Commun..

[25] Wang X., Teng L., Qin X. (2012). A novel colour image encryption algorithm based on chaos. Signal Process..

[26] Linhua Z., Liao X., Wang X. (2005). An image encryption approach based on chaotic maps. Chaos Solitons Fractals.

[27] Zhou Q., Wong K., Liao X., Xiang T., Hu Y. (2008). Parallel image encryption algorithm based on discretized chaotic map. Chaos Solitons Fractals.

[28] Gao H., Zhang Y., Liang S., Li D. (2006). A new chaotic algorithm for image encryption. Chaos Solitons Fractals.

[29] Mao Y., Chen G., Lian S. (2004). A novel fast image encryption scheme based on 3D chaotic bakermaps. Int. J. Bifurc. Chaos.

[30] Etemadi Borujeni S., Eshghi M. (2009). Chaotic image encryption design using tompkins-paige algorithm. Math. Prob. Eng..

[31] Zhang G., Liu Q. (2011). A novel image encryption method based on total shuffling scheme. Opt. Commun..

[32] Wu X., Wang K., Wang X., Kan H. (2017). Lossless chaotic color image cryptosystem based on DNA encryption and entropy. Nonlinear Dyn..

[33] Dong C. (2014). Color image encryption using one-time keys and coupled chaotic systems. Signal Process. Image Commun..

[34] Praveenkumar P., Amirtharajan R., Thenmozhi K., Bosco J., Rayappan B. (2015). Triple chaotic image scrambling on RGB—A random image encryption approach, Security Comm. Networks.

[35] Li C., Lin D., Lü J., Hao F. (2018). Cryptanalyzing an image encryption algorithm based on autoblocking and electrocardiography. IEEE MultiMedia.

[36] Hua Z., Jin F., Xu B., Huang H. (2018). 2D Logistic-Sine-coupling map for image encryption. Signal Process..

[37] Kazim M.A., Naseeruddin M. (1972). On almost semigroups. Alig. Bull. Math..

[38] Mushtaq Q., Yusuf S.M. (1978). On LA-semigroups. Alig. Bull. Math..

[39] Ježek J., Kepka T. (1984). Modular groupoids. Czech. Math. J..

[40] Holgate P. (1992). Groupoids satisfying a simple invertive law. Math. Stud..

[41] Protić P.V., Stevanović N. On Abel-Grassmann’s groupoids (exposition). Proceedings of the Mathematical Conference Pristina.

[42] Mushtaq Q., Yusuf S.M. (1979). On locally associative LA-semigroups. J. Nat. Sci. Math..

[43] Mushtaq Q., Iqbal Q. (1990). Decomposition of a Locally Associative LA-semigroup. Semigr. Forum.

[44] Mushtaq Q., Iqbal M. (1993). On representation theorem for inverse LA-semigroups. Pak. Acad. Sci..

[45] Mushtaq Q., Khan M. (2009). Semilattice decomposition of a locally associative AG**-Groupoid. Algebra Colloq..

[46] Dudek W.A., Gigoń R.S. (2013). Completely inverse AG**-groupoids. Semigr. Forum.

[47] Ruskuc N. (1995). Semigroup Presentations. Ph.D. Thesis.

[48] Yaqoob N., Chinram R., Ghareeb A., Aslam M. (2013). Left almost semigroups characterized by their interval valued fuzzy ideals. Afr. Matematika.

[49] Yaqoob N. (2012). Applications of rough sets to -hyperideals in left almost -semihypergroups. Neural Comput. Appl..

